# Larger Mammalian Body Size Leads to Lower Retroviral Activity

**DOI:** 10.1371/journal.ppat.1004214

**Published:** 2014-07-17

**Authors:** Aris Katzourakis, Gkikas Magiorkinis, Aaron G. Lim, Sunetra Gupta, Robert Belshaw, Robert Gifford

**Affiliations:** 1 Department of Zoology, University of Oxford, Oxford, United Kingdom; 2 Virus Reference Department, Public Health England, London, United Kingdom; 3 Wolfson Centre for Mathematical Biology, Mathematical Institute, University of Oxford, Oxford, United Kingdom; 4 School of Biomedical and Healthcare Sciences, Plymouth University, Plymouth, United Kingdom; 5 MRC-University of Glasgow Centre for Virus Research, Glasgow, United Kingdom; University of California, San Francisco, United States of America

## Abstract

Retroviruses have been infecting mammals for at least 100 million years, leaving descendants in host genomes known as endogenous retroviruses (ERVs). The abundance of ERVs is partly determined by their mode of replication, but it has also been suggested that host life history traits could enhance or suppress their activity. We show that larger bodied species have lower levels of ERV activity by reconstructing the rate of ERV integration across 38 mammalian species. Body size explains 37% of the variance in ERV integration rate over the last 10 million years, controlling for the effect of confounding due to other life history traits. Furthermore, 68% of the variance in the mean age of ERVs per genome can also be explained by body size. These results indicate that body size limits the number of recently replicating ERVs due to their detrimental effects on their host. To comprehend the possible mechanistic links between body size and ERV integration we built a mathematical model, which shows that ERV abundance is favored by lower body size and higher horizontal transmission rates. We argue that because retroviral integration is tumorigenic, the negative correlation between body size and ERV numbers results from the necessity to reduce the risk of cancer, under the assumption that this risk scales positively with body size. Our model also fits the empirical observation that the lifetime risk of cancer is relatively invariant among mammals regardless of their body size, known as Peto's paradox, and indicates that larger bodied mammals may have evolved mechanisms to limit ERV activity.

## Author Summary

Retroviruses have been invading mammalian genomes for over 100 million years, leaving traces known as endogenous retroviruses (ERVs). Early genome sequencing studies revealed a marked difference in the activity of retroviruses among species, with humans largely containing inactive lineages of ERVs, while the mouse contains numerous lineages of active ERVs. We explore the hypothesis that life history traits determine the activity of ERVs in mammalian genomes, and show that larger mammals have fewer ERV copies over recent evolutionary time (the last 10 million years) compared to smaller mammals. This association is determined by body size independently of any confounding variables. We build a mathematical model that shows that ERV abundance in genomes decreases with larger body size and increases with horizontal transmission. Retroviral integration can cause cancer, and our analysis suggests that larger bodied animals control ERV replication in order to postpone cancer until a post-reproductive age. This is in line with a long-standing observation that cancer rates do not fluctuate among mammals of different body size, a phenomenon known as Peto's paradox, and opens up the possibility that larger animals have evolved mechanisms to limit ERV activity.

## Introduction

Mammalian genomes contain large numbers of endogenous retroviruses (ERVs), derived from multiple independent germline invasions over evolutionary time. The human genome contains 31–40 such ERV invasions, termed ‘families’, each derived from a distinct ancestral exogenous retrovirus [1], [2]. These ERVs can continue proliferating after the initial germline invasion until they are inactivated, either through the acquisition of substitutions that occur at the host background level (∼10^−3^ per base per my) or by recombinational deletion [3], [4]. Most ERV families proliferate by reinfection, although some ERVs occasionally switch from reinfecting germline cells to an entirely intracellular life, and this switch can lead to an increase in the size of the ERV family [5]. As a result of these processes, ERVs have come to occupy ∼5–10% of their hosts' genomes [6], [7].

The fixation of a new ERV insertion is influenced by its fitness consequences to the host, and other population genetic parameters [8]; for example a neutral ERV could fix by drift, and a slightly deleterious insertion may hitchhike or fix during a population bottleneck [9]. A small number of ERVs have been exapted and have beneficial functions in their host [10]–[12], but the integration of retroviruses into or near host genes can have highly deleterious effects, as the consequent disruption or alteration of gene expression can lead to malignant transformation [13]. Furthermore, illegitimate recombination between ERVs at different loci can also have deleterious effects, as can the expression of viral proteins. The uncontrolled proliferation of ERVs would therefore be extremely detrimental to their host [14], and this process must be limited either by cessation of replication activity, or by host mediated suppression [15].Vertebrate genomes have evolved a range of responses that act at various stages of the viral life cycle to limit retroviral replication and its associated tumorigenic potential [16], [17].

The diversity and activity of ERVs across mammalian genomes has not been systematically assessed, and it remains unclear what factors have determined ERV abundance in their hosts. Mice and humans, the first two mammalian species to have their genomes sequenced, show strikingly different patterns of ERV activity – most human endogenous retroviruses are inactive, with a striking deceleration in activity over the last 25 million years [7]. In contrast, the mouse genome shows no sign of deceleration in ERV activity and a large number of murine ERVs are active and unfixed in the mouse population [6]. This difference is also reflected in the proportion of catalogued mutant alleles that are due to ERV insertions; ∼10% of mutant alleles can be attributed to ERVs in mice, whereas no such alleles can be attributed to ERVs in humans [18]. It has been suggested that the markedly different ERV activity in human and mouse genomes can be explained by systematic factors in the biology of these hosts [14]. We explore the hypothesis that differences in ERV activity across mammals are determined by differences in host life history, with smaller bodied animals expected to have higher levels of ERV activity. We compare body size with ERV numbers using data from a diverse set of 38 mammals in a multivariate analysis, controlling for confounding variables such as life history traits. We also explore the effect of body size and horizontal transmission on ERV dynamics through a mathematical model. Finally, we discuss our associations of body size and ERV replication in the light of evolutionary theory and cancer biology.

## Results

### Body size has a negative correlation with ERV abundance across mammals

**Figure 1:**
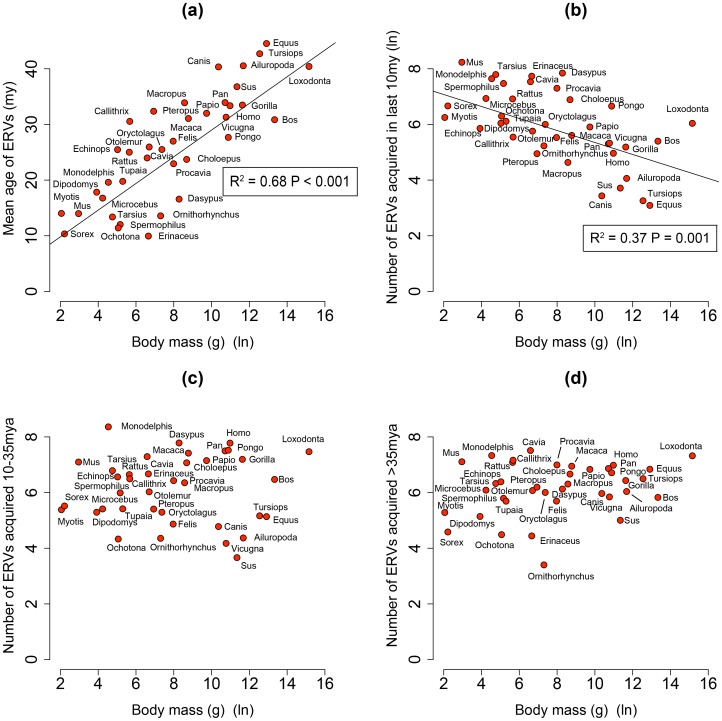
(a) Correlation between mean age of all ERV integrations and body mass from the genomes of 38 mammals. Body mass is log-transformed, and the mean ages are calculated correcting for the substitution rate (R^2^ = 0.68, P<0.001). (b, c, d) The relationship between ERV count and body mass for the number of ERV integrations acquired over the last 10 my, between 10–35 mya and >35 mya in the genomes of 38 mammals (both values log-transformed). The trend lines representing the slope for the regression, corrected for phylogenetic non-independence, and accompanying P-values are plotted. We have taken into account the effect of body size on substitution rate in calculating the ages.

**Figure 2:**
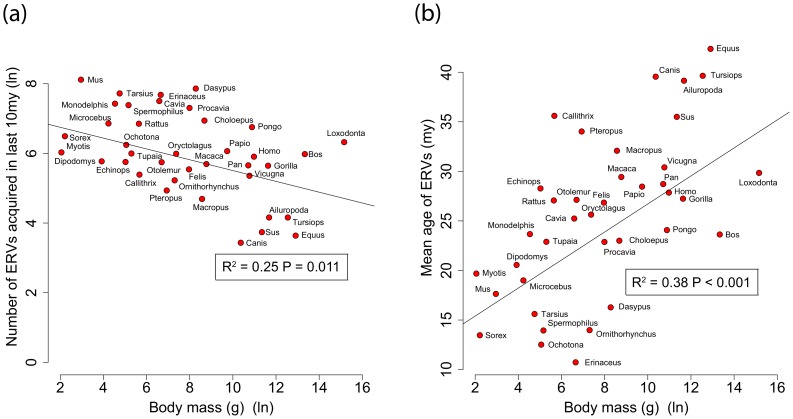
(a) Correlation between ERV count and body mass for the number of ERV integrations acquired over the last 10 my. (b) Correlation between mean age of all ERV integrations and body mass from the genomes of 38 mammals. Body mass is log-transformed, and the mean ages are calculated correcting for the substitution rate. We have not taken into account the effect of body size on substitution rate in calculating the ages.

**Figure 3:**
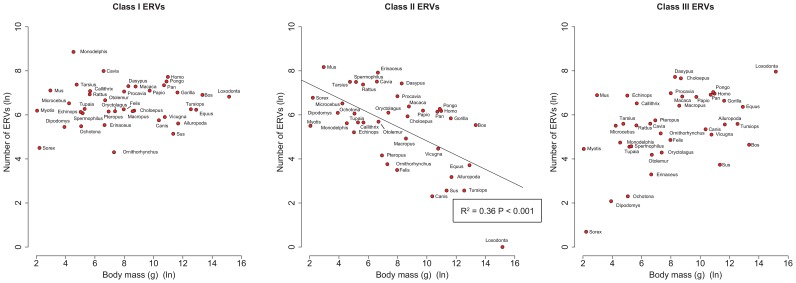
Correlations of number of ERVs against body mass (both log-transformed) by ERV class.

**Figure 4:**
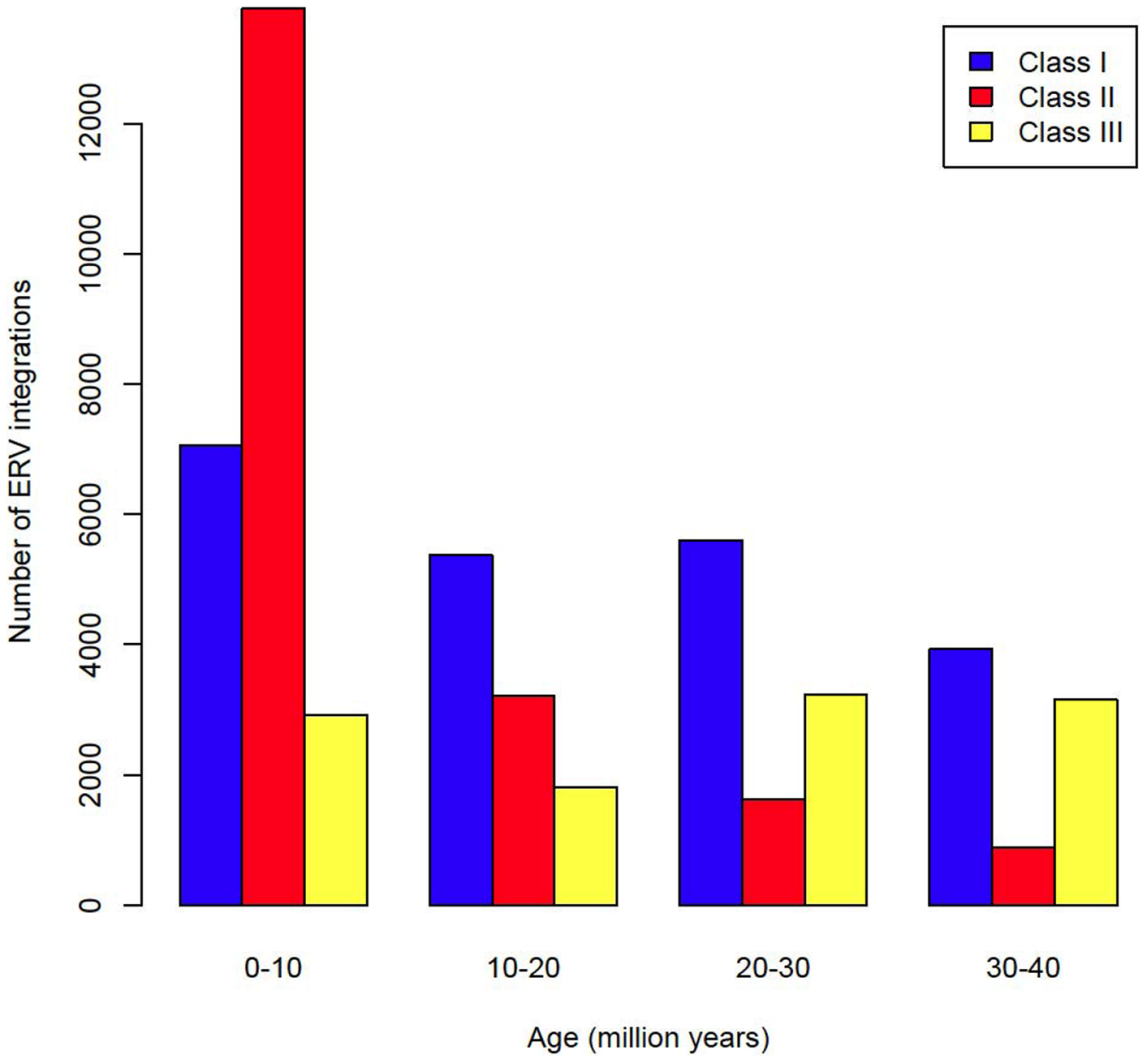
Age distribution of ERVs by class.

By analysing 38 mammalian genomes over approximately the last 10 my period we find a negative relationship between the number of integrated ERVs and body size (Figure 1b, Figure 2a). The correlation is robust if instead of present day body size we use the reconstructed body size at 5 million years ago (P = 0.0069, R^2^ = 0.31), and remains significant if we use a single substitution rate for all mammals (P = 0.01, R^2^ = 0.25). The correlation is dependent on the age of the integration in the genome and is no longer significant when we consider ERVs that are older than 10 my (Figure 1c, Figure 1d). If we exclude ERVs that belong to our previously defined megafamilies [5] with mean divergence <10%, namely the IAP family from *Cavia Porcelus*, the IAP family from *Dipodomys Ordii*, a Class I family from *Felis catus*, and the IAP and ERV-L families from *Mus Musculus*, the correlation remains (P = 0.0042, R^2^ = 0.30). If we split ERVs into their traditional classes, the correlation is significant only for the class II ERVs (Figure 3). There is no data to suggest systematic differences in the biology of retroviruses from different classes, given that the majority of ERVs are derived from extinct retroviral lineages. The three classes differ in their age distribution; class II ERVs are much younger (Figure 4), with the majority of insertions falling within the 10 my era that we use to define the young age category. Thus the observed relationship between body size and Class II ERVs is likely due to their recent replication and not some other difference in their biology such as higher pathogenicity.

### The correlation of body size with ERV abundance is not confounded by another life history trait

Since life history traits are correlated with each other, it is possible that the apparent and inferred correlation of ERVs with body size could be confounded by another trait such as reproductive output (for which gestation period is a proxy) and timing (age at sexual maturity) [19]. The number of mates or the type of placenta might also influence ERV abundance via an increased risk of horizontal or vertical retroviral transmission, respectively. To clarify if number of sexual partners has played a role in determining the number of ERVs per genome, we use testis size as a proxy as it is known to correlate with the number of mates and the strength of sexual selection in mammals [20], [21]. To assess the effect of the placental type we modeled placental invasiveness as a semiquantitative parameter (i.e. marsupials = 1, epitheliochorial = 2, endotheliochorial = 3 and hemochorial = 4) [22]. We evaluated the correlation between ERV integration rate and potential confounders with multivariate models and standard stepwise forward model selection. We included in turn the following confounding variables; time to sexual maturity, gestation period, life span, testis size and placental invasiveness. Body size remained as the only significant variable confirming that it is the only significant predictor of ERV integration rate over the last 10 my (Table 1). The models remain significant when we account for phylogenetic non-independence [23], reconstruct ancestral mass and/or incorporate a body mass dependent substitution rate. Thus, unlike substitution rate [24], [25], ERV integration rate is not a result of shorter generation time. We do not find a significant correlation with testis size, either as an additional predictor variable (P = 0.3) with body size included in the model, or as an interaction term with body size (P = 0.2). Thus, the number of mates does not appear to have played a significant role on the number of ERVs per genome.

**Table 1:** Phylogenetically corrected correlations of number of ERVs per genome acquired over the last 10(log) against life history traits (LHT) confounders.

Univariate analyses			Mutlivariate analyses				
Life traits (LT)	Coefficient	P	LHT confounder coefficient	P	Body Mass (log) coefficient	P	Number of species
Time to sexual maturity (ln)	−0.27	0.15	0.06	0.8	−0.39	0.03	38
Gestation period (ln)	−0.0011	0.45	0.22	0.5	−0.3	0.02	38
Life span (ln)	−0.36	0.13	−0.36	0.12	−0.25	0.04	38
Testis mass (ln)	−0.24	0.01	−0.06	0.78	−0.15	0.2	24
Placental invasiveness	0.34	0.008	0.18	0.12	−0.15	0.02	37

Another possible confounder is the effective population size of the host [26]: species with higher effective population sizes are expected to be more efficient at purging slightly deleterious mutations such as those incurred by ERV proliferation [27]. As a result, since larger bodied animals have smaller effective population sizes [19], [28], we would expect them to have more, not fewer ERVs. Thus, confounding due to effective population size would lead to a correlation in the opposite direction to what we observe, indicating that the observed correlation between body size and ERV numbers is robust against variations in effective population size.

### Relationship between ERV abundance and body size can be explained by a mathematical model of retrovirus-host dynamics

To explore the possible mechanistic links between body size, integration rate and transmission route, we designed a mathematical model (Figure S1). We constructed a compartmental mathematical model using a system of ordinary differential equations to describe the epidemiological dynamics of exogenous and endogenous retroviral infections. There are two broad classes of individuals that need to be considered, the susceptible population 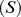 and the infected population. In order to gain a more detailed picture of the latter compartment, namely to elucidate the inter-connected roles between exogenous and endogenous retroviral infections, we further distinguish three infected sub-populations: individuals infected with an exogenous retrovirus 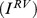, those infected with a single integrated copy of the retrovirus through the process of endogenisation 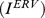, and lastly, those infected with an endogenous retrovirus that has undergone amplification 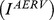.

The overall level of retroviral activity is directly related to the copy number of endogenised retroviruses in the infected population. Since the vast majority of endogenous retrovirus present in the host population persists in the pool of *AERV*-infected individuals, the level of retroviral activity can be represented by the magnitude or size of this compartment. We first explore the roles of three key factors: body size 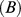
_,_ the rate of retroviral endogenisation 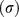 which governs vertical transmission of the retrovirus, and the force of infection 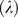 which determines the rate of horizontal transmission of the retrovirus. As shown in Figure S2 increased body size results in a lower number of individuals harbouring amplified endogenous retrovirus when this system reaches equilibrium, (Figure S2, upper plot), while the rate of retroviral endogenisation 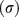 and the force of infection 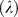 display the opposite relationship (Figure S2, lower plot).

**Figure 5:**
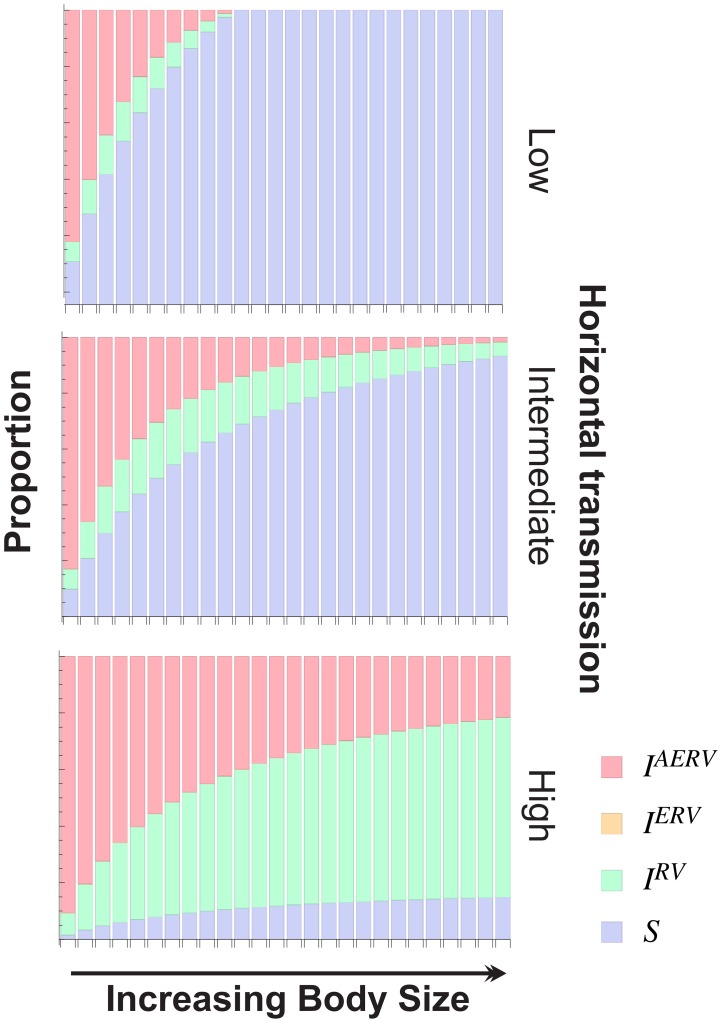
The effect of body size (B) for different horizontal transmission rates (*λ*) on the structure of the population at equilibrium. At high rates of horizontal transmission (*λ*), the proportion of infected individuals reaches a plateau for increasing body sizes. Moreover, in this case, for larger body sizes, there is a greater proportion of exogenous retrovirus infections than there are endogenous ones.

Our model demonstrates that horizontal and vertical transmission are both crucial for the eventual endogenisation and amplification of the retrovirus. If there is no horizontal transmission (i.e. 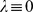), then the retrovirus cannot spread and persist in the population, even if there is a large initial pool of infected individuals (figure 5). Similarly, in the situation where no endogenisation events occur via vertical transmission (i.e. 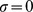), our model shows that infection can become endemic, but remains completely exogenous.

We explored the impact of body size on the structure of the population at equilibrium in relation to the extent of retroviral endogenisation 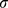 and the force of infection 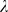 (Figure 5) in more detail. The results in Figure 5 illustrate that higher rates of horizontal transmission, represented by the force of infection 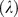, lead to a higher proportion of *AERV* infections for a given body size, and furthermore, highlight our finding that larger body size 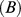 is associated with a lower extent of *AERV* activity, with all other parameters fixed (Figure S2, lower plot). Furthermore, we observe from Figure 5 that for sufficiently high rates of horizontal transmission, the proportion of *AERV* infections plateaus with respect to increasing body size 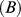. In this case, larger body size is associated with a greater proportion of exogenous infections, as new (exogenous) infections would arise through horizontal transmissions at a faster rate than endogenisation via vertical transmissions. To explore the possibility that the number of horizontal transmissions confound the number of elements per genome, we tested if the number of families per genome is correlated with body size, and find no significant correlation (P = 0.15, R^2^ = 0.08).

## Discussion

We identified 84,223 ERVs, of which 27,711 have integrated in the last 10 my across 38 species of mammal (Table 2). We find that the number of ERV integrations in mammals is negatively correlated to body size. This correlation can explain 37% of the variance in the number of ERV integrations over the past 10 my. We have controlled for confounding variables such as life history and sexual selection, and also confirmed robustness to variation in effective population size. Nevertheless body size can be influenced by other parameters, and it is possible that other factors (e.g. environmental, dietary) contribute to both body size and ERV abundance, thereby explaining part of the remaining variance; for example they might account for the residual variance of outliers (e.g. *Dasypus Novemcinctus* and *Canis familiaris*). Interestingly, *Microcebus murinus*, whose life history evolved rapidly due to its isolation in Madagascar [29], might be expected to be a significant outlier in the correlation, but is very close to the regression line. Perhaps the global distribution and geographic isolation of a species is another determinant of the variance in ERV abundance.

**Table 2:** Reconstructed ancestral body mass and number of ERV integrations in genome sequences.

Species	Common Name	Order	Mass(g)	Mass(g)	Mass(g)	Mass(g)	Total	Loci	Loci	Loci
			Extant	0–10 my	10–20 my	20–30 my	Loci	0–10 my^2^	10–20 my^2^	20–30 my^2^
*Ailuropoda melanoleuca*	Giant panda	Carnivora	118,000	131,600	162,976	227,096	562	62	39	34
*Bos taurus*	Domestic cow	Artiodactyla	618,642	506,826	993,672	2,979,482	1381	386	337	234
*Callithrix jacchus*	Common marmoset	Primates	290	1,361	15,986	47,181	2171	212	221	271
*Canis familiaris^1^*	Domestic dog	Carnivora	31,757	51,162	117,491	218,928	540	31	54	54
*Cavia porcellus*	Guinea pig	Rodentia	728	3,286	6,042	21,061	5109	1802	546	593
*Choloepus hoffmanni*	Hoffmanns two-toed sloth	Pilosa	5,894	14,431	36,676	64,088	2995	1017	624	464
*Dasypus novemcinctus*	Nine-banded armadillo	Xenarthra	3,949	13,459	36,676	64,088	5449	2570	729	1306
*Dipodomys ordii*	Ords kangaroo rat	Rodentia	50	1,396	6,970	22,446	691	320	90	83
*Echinops telfairi*	Small Madagascar hedgehog tenrec	Afrotheria	152	5,647	20,545	52,973	1617	310	391	240
*Equus caballus*	Horse	Perissodactyla	403,599	372,090	439,065	649,462	1140	35	65	75
*Erinaceus europaeus*	West European hedgehog	Erinaceomorpha	778	9,370	51,727	119,255	3039	2137	647	141
*Felis catus^1^*	Domestic cat	Carnivora	2,885	18,927	52,872	169,645	682	252	83	28
*Gorilla gorilla*	Western gorilla	Primates	112,589	77,312	39,124	50,516	2236	271	585	572
*Homo sapiens*	Human	Primates	58,541	50,288	39,124	50,516	3829	348	1085	990
*Loxodonta africana*	African bush elephant	Proboscidae	3,824,540	3,500,961	2,735,801	1,852,642	3820	550	312	780
*Macaca mulatta*	Rhesus macaque	Primates	6,455	9,658	24,536	50,516	3002	286	536	798
*Macropus eugenii*	Tammar wallaby	Marsupialia	5,278	5,257	4,554	6,325	1231	106	176	217
*Microcebus murinus*	Gray mouse lemur	Primates	69	1,210	7,243	29,633	1616	944	134	57
*Monodelphis domestica*	Opossum	Marsupialia	93	197	998	5,610	7481	1647	1537	2158
*Mus musculus*	House mouse	Rodentia	19	154	1,139	11,490	5776	3331	677	389
*Myotis lucifugus*	Little brown bat	Chiroptera	8	475	15,812	72,885	830	408	127	71
*Ochotona princeps*	American pika	Lagomorpha	158	3,059	17,072	44,206	680	513	56	17
*Ornithorhynchus anatinus*	Duck-billed platypus	Monotremata	1,484	6,516	16,581	26,645	294	185	27	43
*Oryctolagus cuniculus*	European rabbit	Lagomorpha	1,591	2,858	15,849	43,846	1003	392	121	69
*Otolemur garnettii*	Northern greater galago	Primates	811	1,647	9,784	34,994	1161	313	143	190
*Pan troglodytes*	Chimpanzee	Primates	45,000	43,518	39,124	50,516	3047	276	690	833
*Papio hamadryas*	Hamadryas baboon	Primates	16,900	14,880	24,536	50,516	2631	422	360	626
*Pongo pygmaeus*	Bornean orangutan	Primates	53,408	47,722	39,124	50,516	3529	843	829	845
*Procavia capensis*	Cape hyrax	Hyracoidea	2,952	32,789	177,092	406,025	3198	1481	346	178
*Pteropus vampyrus*	Large flying fox	Chiroptera	1,028	2,339	45,226	138,581	852	137	89	57
*Rattus norvegicus*	Brown rat	Rodentia	283	251	1,038	11,425	2906	935	396	267
*Sorex araneus*	Common shrew	Soricomorpha	9	1,037	15,391	91,151	1007	650	170	71
*Spermophilus tridecemlineatus*	Thirteen-lined ground squirrel	Rodentia	175	828	3,806	18,775	2339	1594	298	94
*Sus scrofa*	Domestic pig	Artiodactyla	84,472	154,731	618,880	1,710,721	230	42	21	11
*Tarsius syrichta*	Philippine tarsier	Primates	116	9,926	34,791	64,902	3701	2245	445	312
*Tupaia belangeri*	Northern treeshrew	Scandentia	200	1,721	21,772	68,117	923	393	145	59
*Tursiops truncatus*	Bottlenosed dolphin	Cetacea	281,041	646,407	2,268,091	4,418,938	903	55	77	67
*Vicugna pacos^1^*	Alpaca	Artiodactyla	47,500	283,818	919,464	1,723,426	622	210	29	26

1 Body mass data from Canis lupus, Felis silvestris and Vicugna vicugna used for C. familiaris, F. catus and V. pacos respectively

2 Age of ERVs estimated using distance from their nearest neighbour with a substitution rate of 2.2×10^∧^9 substitutions per site per year and a Jukes-Cantor correction for multiple hits

We also see that 68% of the variance observed in the mean age of ERV integrations in a genome (a proxy for recent replication) is explained by body size (Figure 1a), with the number of young (i.e. recently replicating) insertions correlating inversely with body size (Figure 1b) while the number of older insertions do not (Figure 1c, d). These observations suggest that body size limits the number of ERVs in a genome and that the presence of recently replicating ERVs is detrimental to their host. As ERVs accumulate host-induced mutations over time, their activity diminishes until they eventually become neutral. Our study suggests that ERVs that have been active within the last 10 million years could still have moderately deleterious effects, probably in the post reproductive age. Furthermore, the age of an ERV is a good proxy for its pathogenic potential, with pathogenicity decreasing over time. Some ERV families have retained replication capacity for millions of years; HERV-K (HML2) first invaded the primate genome >30 my yet has still been active up until at least ∼500,000 years ago [30], [31]. These recently active ERVs may retain some level of virulence, and therefore still have the potential for malignant transformation [13]. In line with this prediction of intermediate virulence, reconstructed ERVs [32] or recently established present day ERVs [33], [34] have low but detectable viral loads. The presence of pathogenic ERVs in a genome after such a long period of time may appear surprising. It could however be explained by analogy to models of the transmissibility of pathogens within the context of host-parasite co-evolutionary dynamics [35], [36]. Such models incorporate the effects of both transmissibility and virulence on the reproductive success of a parasite, and show that they do not necessarily evolve to be harmless; in some empirical datasets reproductive success is maximised at intermediate levels of both of these parameters [35]. In other words a pathogen can continue to be virulent despite selection imposed by the host for a more benign infection.

We have modelled the spread of retroviruses among hosts and within genomes, distinguishing between exogenous and endogenous retroviruses, and taking into account vertical and horizontal modes of transmission. A key aspect of our model is the assumption that the deleterious effects of a retrovirus in a genome scale with body size. The model shows that as body size increases, the proportion of individuals in the host population that carry ERVs drops (figure S2). Elevated rates of either endogenisation or horizontal transmission lead to higher ERV abundance and accelerate the rate at which ERV abundance increases with body size. For any given rate of horizontal transmission however the overall relationship between body size and ERV abundance is maintained (figure 5). In our model, the body size-associated pathogenic effect of an ERV in a genome is equivalent, whether it has been generated by vertical or horizontal transmission. Horizontal transmission of an ERV would require somatic expression and replication of the virus in order to propagate effectively, which may in turn increase the mortality of the host via a direct result of retroviral infection. Experimental evidence suggests that infections with replication competent retroviruses are more pathogenic when retroviral replication is high (e.g. HIV, or the recently endogenised Koala retrovirus [34], [37]). One way in which the pathogenicity of an ERV can be reduced while its replicative capacity is maintained is through epigenetic regulation in somatic cells. During genomic reprogramming of the germline, transposable elements are expressed and can replicate before being silenced [38], [39], resulting in lower levels of expression in somatic tissues and hence lower transmissibility. Thus, low levels of replication in somatic cells may be favorable for an ERV, enabling it to maximize its own success via vertical transmission while minimizing harm to the host. The association between ERV abundance and body size indicates that somatic replication cannot be completely suppressed and that the pathogenetic effects of ERVs cannot be dissociated from their copy number.

On a macroevolutionary timescale, ERV copy number will be determined both by the number of cross species transmissions and the subsequent proliferation of ERV families. The number of families per genome is orders of magnitude lower than the number of ERVs (mean number of families = 23, mean number of elements = 1073), and most ERVs within a genome come from a small number of families, the so-called superspreaders (or megafamilies) [5]. In line with this uneven distribution of ERVs among families, we do not see a correlation between the number of families within a genome and body size (P = NS, R^2^ = 0.08). Furthermore, ERVs that belong to megafamilies lack the *env* genome that is required for horizontal transmission, highlighting the importance of vertical transmission in determining ERV abundance, despite the ability of ERVs to cross species on timescales spanning millions of years.

Crucially, according to our model the selective cost of an ERV is determined by the body size of the host. Larger bodied animals would be expected to have a higher lifetime risk of cancer as a consequence of having both more dividing cells and longer lifespans. No such association is observed in nature, with relatively invariable risks of cancer in animals with differing body sizes, a phenomenon known as Peto's Paradox [40], [41]. Under our model, the risk of retrovirally induced cancer also scales similarly with body size. The observed negative correlation between body size and ERV integration rate suggests that larger mammals attain a lower ERV virulence cost per body size unit by reducing the number of ERVs in their genome. This should therefore enable them to postpone the onset of cancer until after their reproductive age.

Our results indicate that larger animals exert greater control over ERV proliferation. This could be due to the evolution of mechanisms capable of limiting retroviral activity and consequently limiting the incorporation of ERVs in the genome. Such mechanisms could involve the enhancement of innate or adaptive responses to retroviruses [16], [17], or perhaps epigenetic regulation [42] is more potent in larger mammals. An intriguing alternative is that the effect is indirect via an improved immune surveillance – some genes involved in pattern recognition for defence against pathogens such as viruses are also involved in controlling cancers [43]. Antiviral genes are the result of a continuous and ancient arms race between viruses and their hosts [15], and elucidating their roles in controlling cancer across animals of different body size could provide insights into cancer susceptibility.

## Materials and Methods

### ERV mining and dating of insertions

Our mining of the 38 mammal genomes has been described previously [5], [44], [45]. We estimated age based on the divergence from the most similar other ERV insertion in the same genome (“nearest neighbour”). We favour this approach over cruder metrics that are based on divergence from a consensus sequence, as it takes into account the phylogeny of the ERVs, and over approaches based on divergence between paired LTRs due to the variable quality of the genomes being analysed, most of which do not contain contigs that are long enough to include complete proviral elements. We first calculated nucleotide divergence from the most similar other ERV insertion in the same genome as described in Magiorkinis et al. [5], and then converted this to an integration date assuming a mean nucleotide substitution rate at neutral nuclear protein coding sites in mammals of 2.2×10^−9^ per site per year [46], and corrected for multiple hits using the Jukes-Cantor model. To calculate the average age of ERVs in each genome we took into account the known effect of body size on substitution rate by using a regression of rate against mass with slope of −0.09, i.e. log(adjusted rate) = 0.09×(log(mean mass)−log(mass))+log(unadjusted rate) [47]. We also repeat the correlation between body size and ERV number with a single substitution rate for all mammals.

### Incorporating ancestral body mass

Using the data above we reconstructed ancestral body masses assuming a Brownian motion model of trait evolution as implemented in the package GEIGER in the R language [48]. This program returns the estimated body mass at nodes in the tree, and from these we calculated values at the mid-points of our time intervals (averaging where necessary). We then manually pruned our trees to this point and repeated the regression between number of ERVs and body mass for each time interval, taking the phylogeny into account (Table 2). Our regressions were performed with both present day body size and the reconstructed body size at the mid-point of our time intervals (e.g. body size at 5 million years ago for regression against activity during the last 10 million years).

### Multivariate analysis

Life history traits correlate with each other; for example larger bodied animals tend to live longer and have smaller effective population size [19], [28]. Therefore body size could in principle be a surrogate measure of a different life history trait, as has been previously shown for substitution rate [24]. Mammalian life history data was taken from [49] and the phylogenetic tree from [50]. We collected the testis size for 24 out of 38 species in our study (Table S1). We used the Generalized Least Squares (GLS) approach as implemented by the Analysis of Phylogenetics and Evolution (APE) package [51] in R. We used standard model selection to identify significant confounders of ERV numbers per genome (Table 1).

### A mathematical model of ERV persistence and evolution

Model (1) captures the fundamental dynamics of retroviral infections including the processes of retroviral endogenisation and amplification. The key interactions of the model are illustrated schematically in Figure S1.
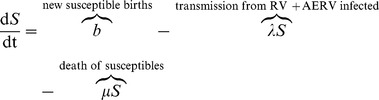


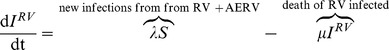


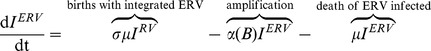


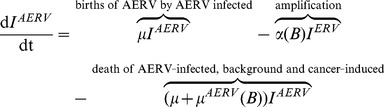
(1)where 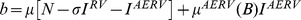 and 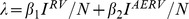.

In model (1), we consider both vertical and horizontal routes of transmission. We also distinguish between exogenous and endogenous retroviral infections. Whereas horizontal transmission can only lead to infection with an exogenous retrovirus (i.e. *RV* compartment), vertical transmission can result in retroviral endogenisation (i.e. *ERV* compartment) and subsequent amplification (i.e. *AERV* compartment).

There are two ways in which new (exogenous) retroviral infections may arise horizontally in an initially susceptible individual, either through contact with an individual infected with an exogenous retrovirus (i.e. *RV* compartment), or alternatively via exposure to an individual infected with an amplified endogenous retrovirus (i.e. *AERV* compartment). We assume that individuals infected with only a single integrated copy of the retrovirus (i.e. *ERV* compartment) are unable to transmit the infection horizontally between hosts. The force of infection 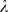 is composed of two terms, 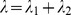, and thus reflects the dual modes of horizontal transmission. There are various different functional forms for the force of infection, and we choose the commonly used form 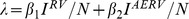, where 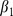 and 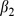 are the respective coefficients of infectious transmissibility for *RV*-infected and *AERV*-infected individuals, and *N* is the total population which is assumed to be constant.

A small proportion 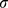, where 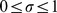, of births from individuals who are infected by an exogenous retrovirus acquire an integrated endogenous copy of the retrovirus, thereby entering the *ERV* compartment. Meanwhile, individuals infected with an integrated endogenous retrovirus (in the *ERV* compartment) undergo retroviral amplification at a rate 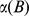, which is dependent on body size 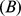. A consequence of retroviral amplification is a greater number of endogenous retroviruses, therefore the size of the compartment of individuals harbouring amplified, endogenised retroviruses is an indirect measure of the overall extent of retroviral activity. Births arising from infected individuals with amplified, endogenous retrovirus (i.e. *AERV* compartment) themselves harbour amplified, endogenous retroviruses.

To investigate the system without unnecessarily over-complicating the dynamical behaviour of the model, we consider a population that is maintained at a fixed size 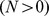 so that 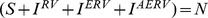. The pool of susceptible individuals is maintained by the birth of new susceptible individuals and is encapsulated in the term 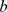 in the 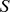 equation, which includes new births of susceptibles from all other compartments as well as a term to balance the in- and out-flux of individuals in the system and ensure that the total population remains constant. We assume that background birth and death rates in each compartment are equal at a constant rate 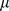. Additional mortality due to the detrimental effects of amplified, endogenous retroviral infection, such as the development of cancer, is reflected in the parameter 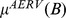, which depends on body size 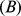. Excess mortality as a consequence of cancer is fed back into the susceptible pool so that, therefore the birth of susceptible individuals can be encapsulated by the term 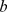, where 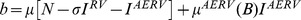


The above discussion highlights an important trade-off between retroviral amplification 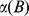, which is beneficial to the long-term persistence of the retrovirus, and increased mortality 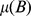 in excess of background death rates as a consequence of the detrimental effects associated with increased retroviral activity. These two factors both depend on body size 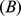, but in opposing ways. Whereas larger body size means increased retroviral amplification, it also results in greater mortality so that both 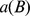 and 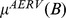 are increasing functions of 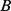. We therefore investigate the role of body size 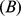 on the outcome of infection. Several additional parameters of significance are the force of infection 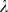 as well as the rate of retroviral endogenisation 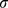 and how varying body size can influence the dynamical behaviour of the infection according to model (1). For the former, we explore how body size can affect the system when differences between the force of infection 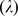 of individuals infected with exogenous retrovirus (i.e. the 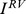 compartment) versus those carrying amplified, endogenous retrovirus (i.e. the 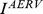 compartment) are taken into account. In terms of the latter, it is expected that a higher rate of endogenisation would result in a greater proportion of individuals with integrated endogenous retroviruses, and we are interested in determining the role of body size with respect to differences in endogenisation rates. Because we have assumed that the total population remains constant, it is sensible to investigate the dynamics of the model with respect to proportions of the total population rather than in terms of the sizes of each compartment.

## Supporting Information

Figure S1 A schematic diagram of the model representing the interactions among four distinct subpopulations: susceptibles (*S*), infected with (exogenous) retrovirus (*I^RV^*), infected with integrated (endogenous) retrovirus (*I^ERV^*), and infected with amplified integrated (endogenous) retrovirus (*I^AERV^*).
(EPS)

Figure S2 The results of model (1) show that the proportion of the population infected with amplified, endogenous retrovirus (i.e. the AERV -compartment) is associated with a larger body size (*B*), and lower rates of endogenisation (*σ*) and force of infection (*λ*). The model also predicts that a higher rate of retroviral endogenisation (*σ*) and a greater force of infection (*λ*) are both linked to a shorter time to reach the endemic steady state.
(EPS)

Table S1 Testis size for 24 species.
(DOCX)
