# Supplementary material for: Larger Mammalian Body Size Leads to Lower Retroviral Activity
Source: PLoS Pathog. 2014 Jul 17;10(7):e1004214. doi: 10.1371/journal.ppat.1004214 (PMC4102558; doi:10.1371/journal.ppat.1004214)
Supplement: Table S1 — Testis size for 24 species. (DOCX) [file ppat.1004214.s003.docx]

| **Species** | **Testis size (g)** |
| --- | --- |
| Bos taurus | 681 |
| Callithrix jacchus | 1.3 |
| Canis familiaris | 27.66 |
| Cavia porcellus | 4.1 |
| Dipodomys ordii | 0.53 |
| Equus caballus | 416 |
| Felis catus | 1.38 |
| Gorilla gorilla | 29.6 |
| Homo sapiens | 50.2 |
| Loxodonta africana | 2300 |
| Macaca mulatta | 76 |
| Macropus eugenii | 31 |
| Microcebus murinus | 2.49 |
| Mus musculus | 0.119 |
| Myotis lucifugus | 10.8 |
| Ochotona princeps | 1.62 |
| Oryctolagus cuniculus | 6.06 |
| Pan troglodytes | 118.8 |
| Papio hamadryas | 27.1 |
| Pongo pygmaeus | 34.2 |
| Rattus rattus | 4 |
| Sorex araneus | 0.28 |
| Spermophilus tridecemlineatus | 1.27 |
| Sus scrofa | 36 |

**References:**

1. Soulsbury CD. Genetic patterns of paternity and testes size in mammals. PLoS One, 2010, 5: e9581

2. Ramm SA, Stockley P.Sperm competition and sperm length influence the rate of mammalian spermatogenesis. Biol Lett. 2010 Apr 23;6(2):219-21.

3. Lemaître JF, Ramm SA, Barton RA, Stockley P.Sperm competition and brain size evolution in mammals.J Evol Biol. 2009 Nov;22(11):2215-21.
